# Temporal dynamics of choice behavior in rats and humans: an examination of pre- and post-choice latencies

**DOI:** 10.1038/srep20583

**Published:** 2016-02-10

**Authors:** Justine Fam, Fred Westbrook, Ehsan Arabzadeh

**Affiliations:** 1 School of Psychology, University of New South Wales, Australia; 2 Eccles Institute of Neuroscience, John Curtin School of Medical Research, Australian National University, Australia; 3 ARC Centre for Excellence for Integrative Brain Function, Australia

## Abstract

Identifying similarities and differences in choice behavior across species is informative about how basic mechanisms give rise to more complex processes. In the present study, we compared pre- and post-choice latencies between rats and humans under two paradigms. In Experiment 1, we used a cued choice paradigm where subjects were presented with a cue that directed them as to which of two options to respond for rewards. In Experiment 2, subjects were free to choose between two options in order to procure rewards. In both Experiments rewards were delivered with distinct probabilities. The trial structure used in these experiments allowed the choice process to be decomposed into pre- and post-choice processes. Overall, post-choice latencies reflected the difference in reward probability between the two options, where latencies for the option with higher probability of reward were longer than those for the option with lower probability of reward. An interesting difference between rats and humans was observed: the choice behavior for humans, but not rats, was sensitive to the free-choice aspect of the tasks, such that in free-choice trials post-choice latencies no longer reflected the difference in reward probabilities between the two options.

According to optimal foraging theory^1^, animals allocate their time and effort across options (such as food sources and mates) in a way that maximizes their returns. Choice behavior shares the same evolutionary roots, where the mechanisms that enable adaptive human choices have evolved from those that maximized fitness in other animals. Identifying similarities and differences in choice behavior across species is informative about how basic mechanisms give rise to more complex processes.

There are clear similarities between animal and human choice behavior, suggesting common underlying mechanisms. For example, animals and humans obey the Matching Law^2^,^3^,^4^,^5^ and favor immediate rewards over delayed rewards^6^,^7^,^8^ (for a review of other similarities see^9^). However, studies of choice have largely focused on choice allocation, and little is known as to how pre- and post-choice behaviors compare between animals and humans. The present study compares pre- and post-choice behavior between rats and humans.

Previous assessments of choice behavior have mainly focused on pre-choice latencies, where an inverse relationship is revealed between reaction times and self-reported choice confidence^10^,^11^,^12^,^13^,^14^. This suggests that reaction times are a cue by which self-reported choice confidence is derived, where short reaction times correspond to high levels of confidence^11^,^12^,^13^,^14^. However, other paradigms show dissociations between outcome expectancy and behavioral measures: this dissociation is evident in the Perruchet effect^15^ which was first demonstrated using classical conditioning of eyeblink responses, but has since been extended across a variety of paradigms^16^,^17^,^18^,^19^.

Outcome expectancy and choice confidence are usually assessed with self-report measures. This typically involves a series of questions after the experimental session, or interspersed sporadically throughout the session. Thus, self-reports may not reflect choice mechanisms on a trial-by-trial basis. We used a discrete-trial structure to parse a single choice into its constituent stages and focused on the temporal dynamics of behavior as manifestations of choice mechanisms. This allowed us to measure response latencies leading up to and following from the explicit choice act. These behavioral correlates of choice were compared between rats and humans, in addition to comparing differences in choice allocation. Our previous work found response latencies to be informative beyond choice allocation as to how environmental contingencies shape mechanisms underlying choice^20^,^21^,^22^. Specifically, we proposed that pre-choice latencies reflected outcome (i.e. reward) expectation, while post-choice behavior reflected choice confidence^20^.

Here, we investigate if the systematic profiles in pre- and post-choice behavior generalize across species under two paradigms: firstly, when choices are cued (Experiment 1), and secondly, when choices are un-cued (Experiment 2). In the cued choice paradigm, a visual cue directed subjects to one of two options which were rewarded probabilistically. This cued-choice paradigm allowed precise temporal alignment of choice latencies, which facilitated interpretation of the differences and similarities between rat and human behavior. This provided a framework with which we could assess response latencies in an un-cued paradigm in Experiment 2, where subjects were free to choose between two options. Importantly, in the present study rats and humans were presented with tasks that were similar in trial structure.

## Experiment 1 Results

**Figure 1:**
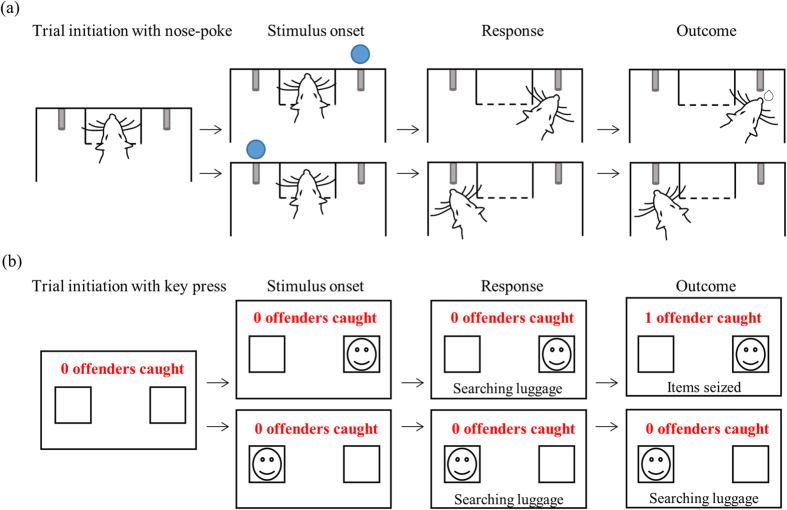
Discrete trial structure of the task is identical for rats and human participants. (**a**) Rats in Experiment 1_R_ initiated a trial by performing a nose poke and maintaining it for a variable delay (stimulus delay; 100–400 ms, uniform distribution), after which the stimulus was presented. The stimulus indicated the location of reward and remained present until a choice was made, which rats indicated by licking either the right or left spout. The stimulus was terminated at first lick contact. If a reward was programmed for the chosen spout, it was delivered after a variable delay provided that rats maintained licking (reward delay; 100–600 ms, uniform distribution). If rats abandoned licking prior to the delay allocated for that trial, then the trial was counted as an unrewarded trial. If no reward was available for the chosen spout (unrewarded trial), there was no external event to indicate the absence of reward and there were no restrictions as to when rats could initiate the next trial. (**b**) For humans in Experiment 1_H_, reward was operationalized as discovering contraband items. Participants initiated a trial by pressing the spacebar, after which a male or female photograph (indicated by the face drawing) appeared on the left or right side of the screen. The photographs remained present until participants pressed the left or right response keys down. Rewards, if available, were delivered after a variable delay (1–5 secs) provided participants kept the response key depressed. Unrewarded trials (either through early termination of a key press response by participants or probabilistically determined), were un-signaled.

Experiment 1 compared choice behavior of rats and humans in a discrete-trial cued-choice paradigm. On every trial, visual cues directed subjects as to which of two reward options to select, and the two options were rewarded with distinct probabilities (three groups: 60–40, 70–30 and 80–20). Figure 1 is a schematic of the task, indicating the identical trial structure for rats (a) and humans (b). We refer to the more frequently rewarded option as High, and the less frequently rewarded option as Low.

We examined two behavioral aspects of choice; firstly the duration between stimulus presentation and response (cue response time), and secondly, the amount of time subjects were willing to wait for a reward (measured as the amount of time that humans spent holding the response keys down: key press duration; or the amount of time rats spent licking at the reward spout: spout sampling duration). Half of the human participants were additionally presented with interleaved free-choice trials, which enabled us to directly assess choice selection and the response latencies associated with that choice.

To examine response latencies, we focused on data from the second and third sessions (second and third quarters) as these periods were when behavior was stable and most sensitive to cue contingencies (Fig 3b–d). The focus on stable choice behavior for analyses is a common approach to address extraneous task effects such as task exploration or boredom which occur early and late in experimental sessions respectively^23^,^24^,^25^. These extraneous task effects were evident in the data for the majority of variables (Supplementary Figs S1–4), particularly in the data from human participants, but is evident in the rat data as well.

### Perception of relative probabilities between cues

**Figure 2:**
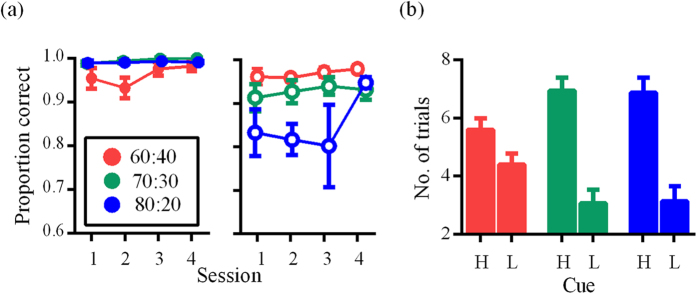
Task performance. (**a**) Group mean proportion of trials where rats correctly followed the stimulus location over the four experimental sessions, shown separately for High (left) and Low (right). For the left panel, overlapping data points are plotted with a slight jitter along the x-axis for visualization. (**b**) Group mean frequency with which the High versus Low cue was chosen on the ten interleaved free-choice trials. Error bars for all figures indicate SEM.

For rats, all groups followed the High cue on more than 90% of trials (Fig. 2a). Performance for Low was also greater than 0.9 for groups 60–40 and 70–30. Group 80–20 showed transient lower performance for the Low cue (0.82 for session 3), which was not evident by session 4. Overall, the behavioral performances indicate effective acquisition of task contingencies where rats followed the cue, even when it provided low probabilities of reward (e.g. 20% probability of reward compared to 0% for not following the cue in an incorrect trial).

Detection of the High versus Low cue probabilities for human participants was verified by examining their response to the interleaved free-choice trials (Fig. 2b). One-way ANOVA revealed a main effect of cue type: across the three probability groups, the High cue was chosen on a significantly greater number of trials than Low (F(1, 42) = 30.738, *p *< 0.001, *η*_*p*_^2^ = 0.423).

### Differences in behavioral latencies: rat data

**Figure 3:**
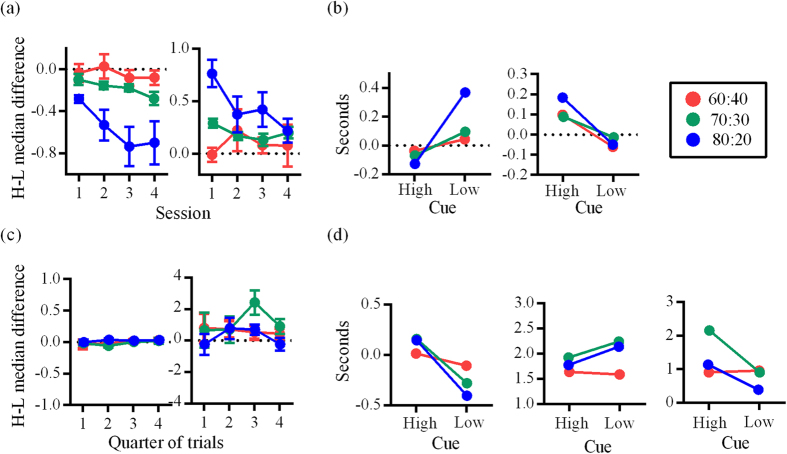
(**a**) Behavioral latencies for rats in Experiment 1_R_. Group means and SEM for the difference between median High and Low cue response times (left) and High and Low spout sampling durations (right) across sessions. (**b**) Group medians for difference from baseline for cue response times (left), and spout sampling durations (right). Baseline was calculated as the median latency of the preceding 10 trials. (**c**) Behavioral latencies for human participants in Experiment 1_H_. Group means and SEM for the difference between median High and Low cue response times (left) and key press durations (right) in session quarters for all trials. (**d**) Left: group medians for difference between key press durations and baseline, which was calculated as the median latency of the preceding 10 trials. Middle: group medians for the difference between free-choice trial cue response times and baseline. Right: group medians for the difference between free-choice trial key press durations and baseline.

Overall, rats were faster to respond to the High cue than the Low cue and this was most pronounced for group 80–20 (Fig. 3a, left). Post-choice durations showed the opposite pattern in that spout sampling durations for High were longer than Low for all groups (Fig. 3a, right). Group 80–20 again showed the greatest difference between High and Low spout sampling durations, but by the fourth session, this became equivalent with other groups.

The pattern observed in Fig. 3a was replicated for the comparison of cue response times and spout sampling durations relative to baseline (Fig. 3b). All groups had significantly longer cue response times relative to baseline when Low was presented and effect sizes reveal that the magnitude of High-Low differences corresponded to the reward probabilities assigned to groups (Mann-Whitney U-test; group 60–40: U = 711568, *p *< 0001, *z *= 9.803, *r *= 0.19; group 70–30: U = 512041, *p *< 0.0001, *z *= 22.242, *r *= 0.42; group 80–20: U = 219042, *p *< 0.0001, *z *= 31.861, *r *= 0.63). Rats showed longer spout sampling durations relative to baseline when responding to High (Fig. 3b) and this was significant for all groups (Mann-Whitney U-test; group 60–40: U = 146955, *p *< 0.0001, *z *= 6.409, *r *= 0.18; group 70–30: U = 124763, *p *< 0.0001, *z *= 6.355, *r *= 0.18; group 80–20: U = 75607, *p *< 0.0001, *z *= 4.52, *r *= 0.14).

### Differences in behavioral latencies: human data

In contrast to what was observed for rats, human participants’ cue response times were not different for the two cues (data points lie around zero) and this lack of a difference between cue response times for High and Low was evident across all four quarters of the experimental session (Fig. 3c, left). Participants’ showed differences in post-choice latencies between High and Low, but this difference varied across the session, where differences in key press durations in the first and last quarter were smaller than in the second and third quarters as reflected in an overall significant quadratic trend (F(1, 171) = 5.648, *p *= 0.02, *ηp* 2 = 0.03; Fig. 3c, right). The U-shaped pattern might reflect task exploration in the first quarter, where participants were learning the range of delays with which reward was presented, and task familiarity in the last quarter, where participants had acquired the knowledge that reward delays were never greater than 5 seconds. This could explain why differences in post-choice latencies in the first and last quarter lie close to zero, in comparison the second and third quarters where post-choice latencies for High were greater than Low.

All groups had longer key press durations when presented with High compared to baseline while key press durations in response to Low were shorter than baseline (Fig. 3d, left). These differences between High and Low key press durations were significant for group 70–30 (Mann-Whitney U test; U = 203211, *p *= 0.019, *z *= 2.34, *r *= 0.06) and group 80–20 (Mann-Whitney U test; U = 175781, *p *= 0.0004, *z *= 3.549, *r *= 0.1), and effect sizes were larger for group 80–20 than 70–30.

For interleaved free-choice trials, data were compared to the median latencies which occurred across the 10 preceding regular trials (choice baseline). In contrast to the similar cue response times for High and Low on regular trials, cue response times on interleaved free-choice trials were significantly longer when participants in groups 70–30 (Mann-Whitney U test; U = 1862, *p *= 0.04, *z *= 2.083, *r *= 0.17) and 80–20 (Mann-Whitney U test; U = 1891, *p *= 0.03, *z *= 2.145, *r *= 0.18) chose Low than when they chose High (for group 60–40 there were no significant difference between High and Low cue response times; Fig. 3d, middle). The opposite pattern was found for key press durations on free-choice trials, where participants in groups 70–30 (Mann-Whitney U test; U = 4624, *p *= 006, *z *= 2.748, *r *= 0.19) and 80–20 (Mann-Whitney U test; U = 9294, *p *= 0.034, *z *= 2.113, *r *= 0.12) showed longer High key press durations than Low (for group 60–40 there were no significant difference between High and Low key press durations; Fig. 3d, right). In addition, median key press durations were longer than choice baseline for both cues for all groups (all medians greater than zero; Fig. 3d, right).

### Relationship between pre- and post-choice latencies

**Figure 4:**
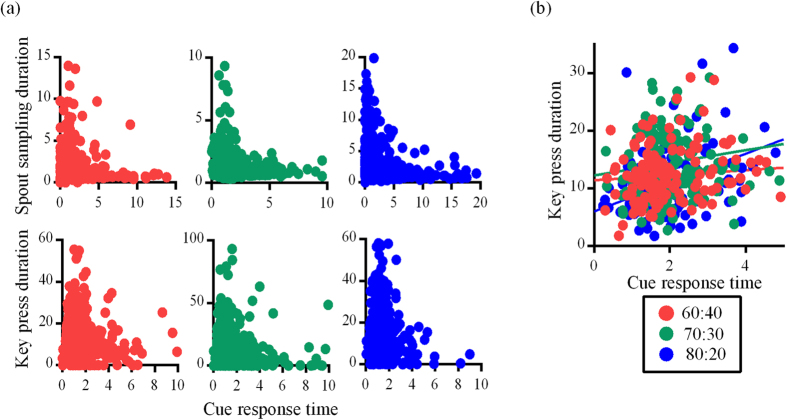
Relationship between pre and post-choice latencies. (**a**) Scatterplots for unrewarded cue response time and spout sampling durations shown separately for each group of rats (top), and unrewarded cue response times and key press durations shown separately for each group of human participants (bottom). Correlations for rats contain between 1300–1400 data points while correlations for human participants contain 850–950 data points. (**b**) Scatterplots for cue response time and key press durations for the ten interleaved free-choice trials (200 data points for each group). All latencies are in seconds.

We used the non-parametric Spearman’s correlation to identify the relationship between pre- and post-choice latencies as the data were not linear. For this, we assessed raw trial latencies and did not use data that were expressed relative to baseline, as the baseline measure was dynamic across the session (median of previous ten trials), thereby introducing variability to the relationship between latency variables. We found cue response times to be negatively correlated with spout sampling durations for all rat groups (group 60–40: r = −0.06, p = 0.0008; group 70–30: *r *= −0.15, *p *< 0.0001; group 80–20: *r *= −0.12, *p *< 0.0001; Fig. 4a, top). For human participants the correlation between cue response times and key press durations was not systematic and was positive or negative depending on the contingencies (group 60–40: *r *= 0.05, p = 0.044; group 70–30: r = −0.08, *p *= 0.001; group 80–20: r = 0.21, p < 0001; Fig. 4a, bottom). In contrast, there was a significant positive relationship between cue response times and key press durations for all groups on interleaved free-choice trials (group 60–40: *r *= 0.18, *p *= 0.035; group 70–30: *r *= 0.24, *p *= 0.003; group 80–20: *r *= 0.38, *p *< 0.0001; Fig. 4b). Supplementary Table S5 additionally shows how correlation coefficients developed across each session (for rats) or session quarter (for human participants).

## Experiment 1 Discussion

Rats and humans showed similar patterns of behavioral latencies. This was clearest for the post-choice response of spout sampling durations and key press durations; both rats and humans spent a longer duration of time responding when presented with the High cue on unrewarded trials compared to the Low cue. The cue response times were longer for Low compared to High and this was true for rats and the interleaved free-choice trials for humans. In addition, the same negative relationship between pre- and post-choice latencies were seen for both rats and humans, but for human participants, this relationship was reversed on interleaved free-choice trials.

The free-choice trials influenced choice behavior. Key press durations for Low were above baseline for interleaved free-choice trials, but below baseline for the regular trials. This contradictory pattern of responding may relate to the choice aspect in free-choice trials: in such trials, participants’ choices might have reflected an expectation about which cue would be most likely to be rewarded (given the experience of the 10 preceding trials). Thus, we propose that participants selected the Low cue on free-choice trials when they felt it was more likely to rewarded, and this manifested as longer key press durations. Alternatively, it might be the case that participants viewed free-choice trials as an opportunity for task exploration. A related possibility might be that free-choice trials provided a means of testing the experienced probabilities on the 10 preceding trials. In both these possibilities, participants’ choices would be motivated by uncertainty resolution as distinct from outcome expectancy.

Experiment 2 used an un-cued choice task to verify the effect of self-generated choice on post-choice latencies. Experiment 2 also aimed to verify that the same positive relationship between cue response times and key press durations would be evident when all trials involved a free-choice for human participants. To do this, we presented human participants with an un-cued version of the task in Experiment 1. For rats, this un-cued task is identical to that used in our previous work and we re-analyzed the data from that study^20^.

## Experiment 2 Results

**Figure 5:**
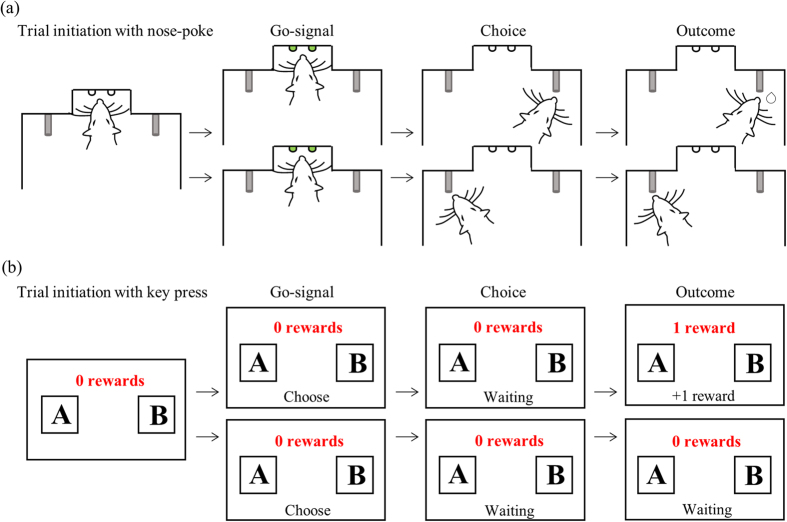
Discrete trial structure of the un-cued task is identical for rats in Experiment 2_R_ (**a**) human participants in Experiment 2_H_ (**b**). Briefly, 36 experimentally naïve, adult, male Wistar rats were presented with a choice on every trial between a left and right reward spout. As in the first experiment, rats initiated a trial by performing a nose poke and maintaining it for a variable delay (100–600 ms, uniform distribution). Two diode lights located in the middle of front wall of the nose poke aperture were lit after this delay to indicate the go-signal. The lights remained on until a choice was made. For human participants, a trial was initiated by pressing the space bar. A go-signal, appearance of the word ‘Choose’, was displayed on the computer screen after a short variable delay (0.5–2 secs). Participants then selected between A or B by making a key-press response and maintaining it for as long as they wanted. Rewards were delivered after another variable delay (1–5 seconds, uniform distribution). There was no external signal to indicate when trials were unrewarded.

In Experiment 2, we presented subjects with an un-cued version of the first experiment (see Fig. 5 for task details). The trial structure was identical to that of Experiment 1, and the same reward probabilities were used (60–40, 70–30 and 80–20). Pre-choice behavior was defined as choice execution latency. This was calculated as the duration of time between onset of the go-signal and onset of the choice response (first lick at a reward spout for rats; depression of a response key for humans). Post-choice latencies on unrewarded trials were defined in the same way as in Experiment 1: spout sampling durations for rats and key press durations for human participants.

### Choice allocation

**Figure 6:**
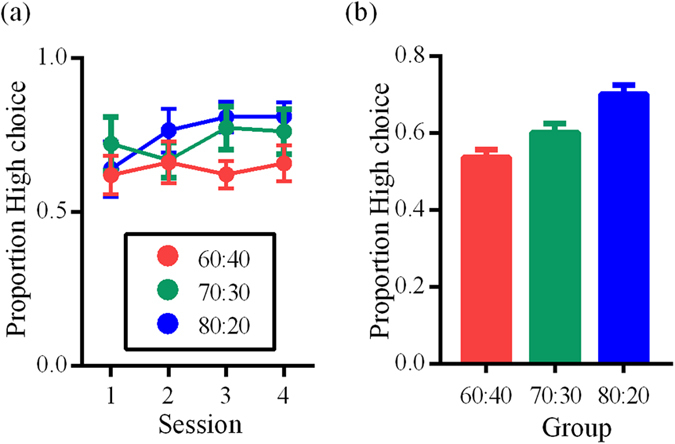
Group mean proportion of High choices for rats across the four experimental sessions (**a**) and humans participants (**b**). Error bars indicate SEM.

Rats and humans selected High for the majority of trials (all High choice proportions greater than 0.5, Fig. 6), indicating that they were sensitive to the reward probability manipulations. For both rats and humans, differences in High choice proportions reflected the reward probabilities; group 80–20 selected High the most, while group 60–40 the least.

### Difference in behavioral latencies: rat data

**Figure 7:**
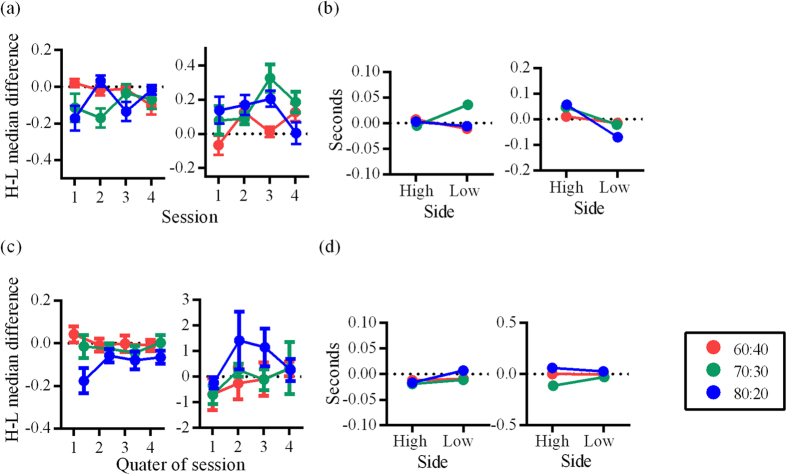
(**a**) Behavioral latencies for rats in Experiment 2_R_. Group means and SEM for the difference between median High and Low choice execution latencies (left) and spout sampling durations (right) across four sessions. (**b**) Group medians for difference between choice execution latencies and baseline (left), and spout sampling durations and baseline (right), shown separately for High and Low. (**c**) Behavioral latencies for human participants in Experiment 2_H_. Group means and SEM for the difference between median High and Low choice execution latencies (left) and key press durations (right) in session quarters for all trials. (**d**) Group medians for difference between choice execution latencies and baseline (left), and key press durations and baseline (right), shown separately for High and Low.

Overall, a comparison of High and Low choice execution behavior revealed longer latencies for Low than High and this is consistent with Experiment 1_R_. However, there was some variability in this respect in early sessions which were no longer evident by session 3 (Fig. 7a, left). To maintain consistency with previous analyses, we focused on the second and third sessions for further trial-by-trial analysis. Spout sampling durations also showed early variability in the pattern of differences between High and Low sides (Fig. 7a, right). By the second session, all groups showed longer median High spout sampling durations.

When choice execution latencies from the second and third session were examined relative to baseline, small but significant differences were found that were not consistent across groups (for groups 60–40 and 80–20, latencies were longer relative to baseline for High than Low; Mann-Whitney U test; group 60–40: U = 728502, *p *< 0.0001, *z *= 4.027, *r *= 0.08; group 80–20: U = 655230, *p *< 0.0001, *z *= 4.337, *r *= 0.08; while the opposite pattern was observed for group 70–30; U = 668175, *p *< 0.0001, *z *= 10.072, *r *= 0.19; Fig. 7b, left). This is different to what was observed in Experiment 1_R_, where Low cue response times were longer than baseline, while High cue response times were shorter than baseline. In contrast, all groups showed the same pattern of differential spout sampling durations; High spout sampling durations were longer than baseline while Low spout sampling durations were shorter than baseline. This was significant for group 70–30 (Mann-Whitney U test; U = 77649, *p *= 0.006, *z *= 2.739, *r *= 0.09) and group 80–20 (Mann-Whitney U test; U = 48532, *p *< 0.0001, *z *= 4.194, *r *= 0.16; Fig. 7b, right).

### Difference in behavioral latencies: human data

Participants in groups 60–40 and 70–30 did not modulate their choice execution latencies according to the High/Low status of options (Fig. 7c,d). Although there was a trend for Low choice execution latencies to be longer than High choice execution latencies, this was only significant for group 80–20 (Mann-Whitney U test; U = 871743, *p *< 0.0001, *z *= 4.857, *r *= 0.09; Fig. 7d, left). Choice execution latencies were shorter than baseline (with the exception for the Low option for group 80–20; data points lie below zero in Fig. 7d, left). This indicates that choice execution latencies decreased across the session as participants became more familiar with the temporal structure of the task. This within-session decrease was small, however, as it is not apparent as a linear trend when data were examined in session quarters and not relative to baseline (Fig. 7c, left).

Key-press durations for High versus Low showed no differences (Mann-Whitney U test; group 60:40: U = 172180, *z *= 0.05, *p *= 0.96, *r *< 0.01; group 70–30: U = 140677, *z *= 0.84, *p *= 0.40, *r *= 0.03; group 80–20: U = 145702, *z *= 1.78, *p *= 0.075, *r *= 0.05; Fig. 7d, right). This is in contrast to what was observed in Experiment 1 and in-line with our hypothesis.

### Relationship between pre- and post-choice latencies

**Figure 8:**
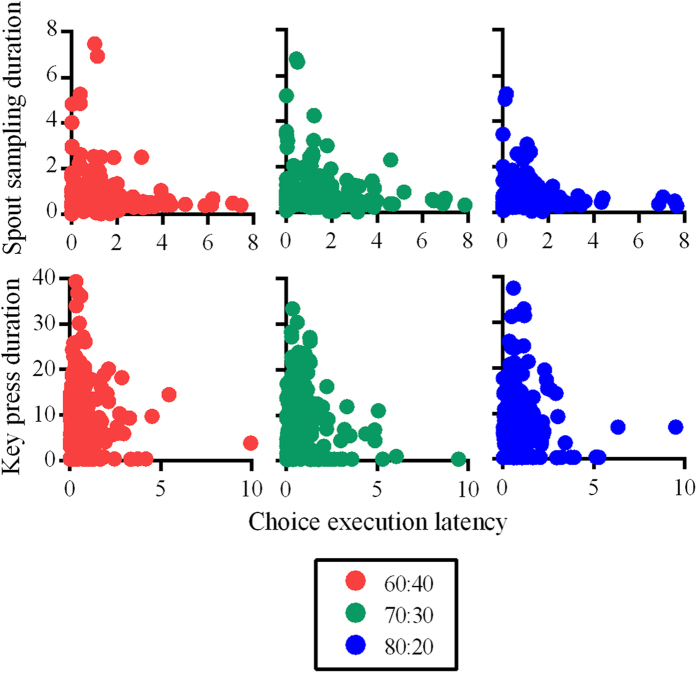
Relationship between pre and post-choice latencies. Scatterplots for unrewarded choice execution latencies and spout sampling durations shown separately for each group of rats (top), and unrewarded choice execution latencies and key press durations shown separately for each group of human participants (bottom). Correlations for rats contain between 800 and 900 data points while correlations for human participants contain 700 to 800 data points. All latencies are in seconds.

We again used Spearman’s correlation for this analysis for both rats and humans as the data were not linear. For rats in Experiment 2_R_, the relationship between choice execution latencies and spout sampling durations were negatively associated for group 70–30 (group 70–30: *r *= −0.17, *p *< 0.0001; Fig. 8, top), but positively associated for group 80–20 (group 80–20: *r *= 0.08, *p *= 0.0009; Fig. 8, top). For human participants, there was a positive relationship between choice execution latencies and key press durations. This replicates what was found for interleaved free-choice trials in Experiment 1_H_. This was significant for groups 70–30 and 80–20 (group 70–30: *r *= 0.15, *p *< 0.0001; group 80–20: *r *= 0.09, *p *= 0.0007; Fig. 8, bottom).

## Experiment 2: Discussion

Choice proportions indicated that rats and humans detected the greater likelihood of reward from High compared to Low. This is particularly interesting for human participants, as we found no difference in key press durations between High and Low. Hence, although the differences in reward probabilities were reflected in choice allocation, they did not manifest in post-choice commitment. This can be seen to be in-line with what we observed in Experiment 1. From Experiment 1_H_, we found that human participants showed an increase in choice commitment for both High and Low on interleaved free-choice trials, in that key press durations were elevated from baseline for both the Low and High cue (all data points lie above zero). Thus, choices which are self-generated result in an escalation of commitment. Experiment 2_H_ is a further demonstration of escalation of commitment using an un-cued task where every trial consisted of free-choice between High and Low. In this second experiment, escalation of commitment was evident in equivalent key press durations for High and Low. The positive relationship between pre-choice latencies and key press durations seen in Experiment 1_H_ was also replicated in 2_H_, where long choice execution latencies were associated with long key press durations. Thus, for human participants, key press durations did not reflect the differences in reward probabilities between High and Low, but instead correlated to the duration of choice execution.

By contrast, rats in Experiment 1_R_ and 2_R_ showed consistent spout sampling duration patterns reflecting greater choice commitment for High than Low regardless of whether choices were cued or not. Thus, while the free-choice aspect changed the appearance of choice commitment in human participants, it did not alter the choice behavior of rats. For rats in Experiment 2_R_, the correlation between choice execution latencies and spout sampling durations was not consistent, suggesting that spout sampling durations reflected the differences in reward probabilities between High and Low, and were not related to the amount of time taken to execute a choice.

## General Discussion

The present study examined choice behavior of rats and humans using a discrete trial procedure where rewards were delivered probabilistically. In Experiment 1, rats and humans were cued as to which option to select, and we quantified post-choice behavior as spout sampling durations for rats, and key press durations for humans. For both rats and humans, pre-choice latencies were quantified as cue response times. Detection of reward contingencies was additionally assessed for half of the participants through free-choice trials interleaved throughout the session. These free-choice trials were unrewarded and allowed us to directly compare participants’ choices, cue response times and key press durations. We found that behavior on these free-choice trials differed from regular trials and this was examined further in a second experiment which employed an un-cued version of Experiment 1.

While there was no direct indication of rats’ cue preference as an index of learnt reward contingencies, the high proportion of correct trials indicated that rats experienced the allocated proportion of rewarded and unrewarded trials for each reward spout. Cue response times and spout sampling durations also showed marked group differences which corresponded to group reward probabilities. In addition, choice allocation in Experiment 2_R_ indicated that rats had learnt there was a greater probability of reward from High. Rats showed the same pattern of differential latencies between cued and un-cued choice. This was clearest for spout sampling durations, which were longer for High than Low regardless of whether choices were cued or not. For group 60–40 in Experiment 2_R_, choice proportions were at equivalence (0.5) but there was still evidence of differential spout sampling durations in some of sessions (Fig. 7b, right). Overall, pre-choice latencies for High were shorter compared to Low, (Fig. 3a, left and 7a, left). The further analysis of pre-choice latencies relative to baseline revealed inconsistent effects for Experiment 2_R_ (Fig. 7b, left), however, the greater accuracy in temporal alignment through the use of a cue resulted in clearer differences in cue response times between groups (Fig. 3b, left).

Across both experiments, human participants were also sensitive to the reward probability manipulations, as reflected in the greater proportion of High choices for Experiment 2_H_ and the interleaved free choice trials in Experiment 1_H_. This was the case even for group 60–40. Thus, differences in behavioral latencies between rats and humans were not due to a lack of perception of reward contingencies. Overall in Experiment 1_H_, key press durations reflected the High-Low differences in reward probability, in that High key press durations were longer than Low. Differential key press durations on regular unrewarded trials also reflected group assignment, where the greatest difference was seen for group 80–20. Differential key press durations showed another interesting pattern: in Experiment 1_H_, key press durations on regular trials for High were longer than baseline, while key press durations on regular trials for Low were lower than baseline. This pattern was also seen for free-choice trials but with an additional upward shift, such that Low key press durations were also increased from baseline.

One difference between rat and human choice behavior was that cue response times for human participants in Experiment 1_H_ (excluding free-choice trials) did not vary with reward probability, while for rats in Experiment 1_R_, cue response times reflected the difference in reward probabilities. This is interesting as the knowledge that High was more frequently rewarded did manifest in key press durations of human participants. Another difference between rat and human behavior was the way free-choice altered choice behavior for humans, but not for rats. This was observed in two ways. Firstly, for humans, self-generated choice in Experiment 2 resulted in equivalent commitment levels for High and Low (equivalent key press durations in Experiment 2_H_), but rats showed longer spout sampling durations for High than Low when choices were cued and un-cued. Secondly, the choice aspect of free-choice trials in Experiment 1_H_ and the un-cued task in Experiment 2_H_ resulted in a positive relationship between pre-choice latencies and key press durations for human participants, as indicated by the correlation analyses. One possibility is that for human participants, longer pre-choice latencies reflected the need for deliberation due to uncertainty as to which was the better option. In order to resolve this uncertainty, participants invested more time in determining outcomes by holding the response key down for durations that exceeded the maximum reward delay. By contrast, self-directed choice for rats resulted in more variable relationships between choice execution latencies and spout sampling durations. For rats in the cued choice task, there was a negative relationship between pre-choice latencies and post-choice latencies. This could reflect that the same process of learned value which shortens approach latencies, also increases expectation of reward and increases sampling durations. This supports previous research suggesting that response latencies can reflect choice confidence. It is important to note, however, that these correlations may also point to other explanations. Indeed, although the *p* values associated with the correlation analyses are highly significant (all *p* < 0.01), the *r* values themselves are small, pointing to small but robust effects that could indicate a relationship with a third variable (e.g. passage of time since commencement of the experiment).

Most characterizations of confidence relate to self-report measures. Behavioral measures of choice confidence have recently been used in a variety of paradigms^26^,^27^,^28^. Waiting times, the duration of time subjects invest in waiting for reward, is a validated behavioral index of choice confidence similar to the measures of spout sampling durations and key press durations used here^27^. In the present study, the amount of time subjects spent responding for a reward is at the expense of initiating a new trial and potentially obtaining more rewards. Hence, the length of this waiting time is a direct reflection of subjects’ reward expectancy. The behavioral latencies used in the present study can bridge the gap between animal and human studies of choice confidence because the same behavioral measures can be assessed across species to identify the basic mechanisms which underlie choice. Our observation that rats and humans invest more time for High is in-line with optimal foraging theory. When reward contingencies are unknown (such as in the present experiment), behavioral strategies are acquired through interaction with the environment and experience with reward likelihood. In the current study, direct experience with the greater probability of reward from High resulted in the behavioral strategy of longer spout sampling durations and key press durations for rats and humans.

We were able to show that post-choice latencies can be dissociated from the explicit choice itself. Recent studies of the Perruchet effect have similarly shown that reaction times can be dissociated from self-reported outcome expectancy using human participants^16^,^17^,^18^,^19^. This has led to a suggestion that meta-cognitive processes are distinct from automatic reflex-like behaviors. Here, we found such dissociations in rats as well as humans. The trial structure in this study can be used to elucidate this further, and identify the relationship between these various components of choice across species. For example, it would be possible to identify which aspect of choice behavior adapts quickest to changes in environmental probabilities. We previously found that rats adjusted their spout sampling durations to changes in reward probabilities between High and Low prior to adjusting their choice proportions^20^,^21^,^22^. This indicates that the information gathered during the post-choice period can guide changes in choice allocation.

We conducted four experiments to compare choice behavior in rats with humans. For both humans and rats immediate post-choice behavior demonstrated greater commitment to the more frequently rewarded option. We identified that the element of free-choice resulted in an escalation of commitment for humans, which manifested as equivalent key-press durations for High and Low in the second experiment, despite the difference in reward probabilities. This was not observed for rats; instead the difference in High versus Low reward probabilities exerted control over post-choice latencies across both experiments, when choices were either cued or un-cued. Overall, rat and human choice behavior followed optimal foraging theory in that, suggesting a common mechanism which guides choice across species.

It is important to note that the findings presented here are open to interpretation. Further research is needed to verify the underlying mechanisms proposed here, as well as to rule out the alternative explanations that have also been put forth.

## Methods

### Experiment 1

#### Experiment 1_R_

##### Subjects

Subjects were 18 experimentally naïve, adult, male Wistar rats with initial weights of 266–575 g. Rats were housed in a climate controlled colony room on a 12 hour light-dark cycle (lights on at 7 am). All procedures were approved by, and carried out in accordance with the guidelines provided by, the Animal Care and Ethics Committee at the University of New South Wales. Rats were given one hour free access to food and water after each session and body weights were monitored to ensure they did not drop below 85% of their free-feeding weight.

##### Apparatus and stimuli

Rats were trained in a clear Plexiglass chamber measuring 30 cm (length) × 30 cm (width) × 50 cm (height) with a circular nose-poke port (5.5 cm long × 3.5 cm wide, extending 4 cm into the chamber). The nose-poke port had optical sensors attached to the inside walls to detect head entries. To the right and left of the aperture were drinking spouts which allowed a 500 ms delivery of 5% sucrose solution via pumps located outside the testing chamber. These reward spouts were fitted with sensors to detect licking at a temporal precision of 1 ms. An infrared camera provided video monitoring of behavior. The experiment was controlled using programs written in Matlab. All training occurred in a dark room with background noise (~70 db) to mask any extraneous sounds. Visual stimuli (a white circle, 2.5 cm in diameter) were presented directly above the left or right reward spouts depending on the cue allocation on every trial.

##### Design and task

Figure 1a illustrates the structure of each trial. Each experimental session consisted of at least 250 trials, and the stimulus had 50% probability of being presented at the left and right spouts. Across all three groups, the number of left and right trials were identical (minimum of 125 left and right), however the frequency with which those trials were rewarded varied according to three probability pairs: 60–40, 70–30 and 80–20; counterbalanced for position (left or right) within groups.

##### Procedure

###### *Spout shaping*

 Rats were placed in the experimental chamber for 10 minutes and reward was freely available from both reward spouts. The circle stimulus was presented simultaneously with every reward delivery. The nose-poke aperture was blocked at this stage.

###### Nose-poke and stimulus shaping

 The nose-poke aperture was un-blocked and stimuli were presented only when rats had performed a nose-poke. Across four sessions, stimulus and reward delays were introduced and gradually increased: 0 ms (session 1), 75 ms, 100–200 ms, 100–400 ms (session 4). Training continued until rats were correctly following the stimulus location on more than 90% of trials.

###### Training

 Rats were presented with the assigned reward probabilities for four sessions.

#### Experiment 1_H_

##### Participants

Participants were 90 undergraduate students (20 male, mean age = 20.3 years) enrolled at the University of New South Wales who volunteered to participate in the study in return for course credit. All participants provided informed consent prior to commencement of the experiment. All procedures were approved by, and carried out in accordance with the guidelines provided by, the University of New South Wales Human Research Ethics Committee. Participants were randomly allocated to one of three conditions.

##### Apparatus and stimuli

Participants were presented with five female and five male photographs (each presented 20 times) in random order over a total of 100 trials. Photographs were selected from the Glasgow Face Matching Test database (www.abdnfacelab.com/downloads). Gender of the photographs was associated with reward probability; for example, male photographs had a higher probability of reward compared to female photographs for some participants and this was counterbalanced across groups. We used a range of male and female faces to increase the level of engagement with the task. The computer task was controlled using programs written in Matlab (The Math Works, Inc.).

##### Design and task

Participants were told to imagine they were employed by the customs department at the airport to search people’s luggage for contraband items (reward). Participants were also told that they were free to determine the duration of time spent searching a particular person’s luggage, however, not every person they encounter carries contraband items. This therefore presented participants with the task of having to determine the balance between searching long enough (so as not to miss finding contraband items) yet not too long (thereby delaying the entire task).

Photographs were presented either on the left or right of the computer screen and participants responded by pressing and holding down the F1 key (labeled “left”) for photographs presented on the left, and the F10 key (labelled “right”) for photographs presented on the right. Release of either key terminated a search and the trial; the key had to be pressed down continuously for the duration of time that participants wanted to engage in the luggage search. The order of left/right location of stimuli was randomly determined and half of each stimulus type (male/female) were presented on the left and right. Participants were given feedback as to the number of total offenders they had successfully apprehended through a tally shown on the top of the computer screen, but this tally did not show the breakdown of contraband items that had been obtained from male versus female photographs.

The experiment was a between subject-design, with three groups which differed in the probability of reward associated with male and female stimuli; 60 vs 40; 70 vs 30; and 80 vs 20 (counterbalanced across gender of stimuli). The key press durations on unrewarded trials (when contraband items were not found) were the focus of our analyses and we do not consider key press durations when contraband items were found.

Half of the participants in each probability group additionally experienced interleaved free-choice trials where a male and a female photograph appeared simultaneously (left or right counterbalanced), and a choice had to be made by pressing the corresponding response key. Free-choice trials occurred every 10 regular trials and were all unrewarded. The free-choice trials assessed whether participants were sensitive to the reward probability manipulations.

##### Procedure

Demographic information and written consent was obtained before the experiment commenced. Participants were given written instructions for the task and asked to complete the task with one hand only (dominant hand). We did not identify any systematic differences related to handedness of the participant. Participants were then given a full debriefing and the experiment concluded.

### Experiment 1 analysis

The experimental task used for Experiment 1_R_ and 1_H_ differ in terms of the stimulus property that predicted rewards. For rats in Experiment 1_R_, the stimulus location was associated with reward probability, thus there was a High side versus a Low side. By contrast, the gender of presented stimuli was associated with reward probability for human participants in Experiment 1_H_; rather than a High side/Low side, participants had to distinguish between male and female photographs. This difference did not alter the task structure between rats and humans, and Fig. 1 depicts the identical temporal alignments within a trial.

All analyses were carried out using Matlab, Graphpad and SPSS. Cue response times were defined as the duration of time between onset of stimulus and onset of response (spout licking for Experiment 1_R_, key press onset for Experiment 1_H_). Post-choice latencies on unrewarded trials were defined as the duration of the response; for Experiment 1_R_ this was the duration between first and last lick at reward spouts (spout sampling duration), while for Experiment 1_H_, this was the duration with which participants held the response key down (key press duration).

We compared behavior towards High and Low by taking the difference between High and Low median latencies for each individual rat or participant. These difference scores reflect differences within individuals in the behavior towards High and Low, which might be obscured by between-subject variability if assessing absolute latencies across groups. Descriptive statistics for latency variables are shown in Supplementary Tables S1–4. This table also indicates the skew in the distribution of the latency variables, which warrants non-parametric analyses.

### Experiment 2

#### Experiment 2_R_

Additional analyses of the data from our previous study were carried out for comparison with the human behavior^20^. The analyses carried out in this study do not overlap with those in our previous work. The method is detailed in our earlier study^20^ and is illustrated in Fig. 5. Briefly, rats initiated a trial by nose-poke, received a go signal, and chose between two reward spouts. Reward spouts had independent reward probabilities, therefore, on any given trial, reward could be available on one, both, or neither of the spouts. The same probability pairs in Experiment 1 were used: 60–40, 70–30 and 80–20 (counterbalanced for left/right position within groups).

#### Experiment 2_H_

##### Participants

Participants were 60 undergraduate students (14 male, mean age = 21.2 years) enrolled at the University of New South Wales who volunteered to participate in the study in return for course credit. All participants provided informed consent prior to participating in the experiment. All procedures were approved by, and carried out in accordance with the guidelines provided by, the University of New South Wales Human Research Ethics Committee. Participants were randomly allocated to one of three conditions (n = 20; see Design). Apparatus, stimuli, and procedure were similar to Experiment 1_H_.

##### Design and task

Participants completed a computer task which closely resembled that described in Experiment 1_H_. The key difference was that participants were instructed to make a choice between a left or right option (A or B) on every trial. The two options were rewarded according to the same probability pairs (60:40; 70:30; 80:20), and required the same key-press response as in Experiment 1_H_. A tally of rewards received was presented on the computer screen, but did not provide a breakdown of the number of rewards received from selecting A or B. This maintained the requirement for participants to acquire internal representations of which option was more frequently rewarded.

## Additional Information

**How to cite this article**: Fam, J. *et al*. Temporal dynamics of choice behavior in rats and humans: an examination of pre- and post-choice latencies. *Sci. Rep.* **6**, 20583; doi: 10.1038/srep20583 (2016).

## Supplementary Material

Supplementary Information
