# Supplementary material for: Temporal dynamics of choice behavior in rats and humans: an examination of pre- and post-choice latencies
Source: Sci Rep. 2016 Feb 10;6:20583. doi: 10.1038/srep20583 (PMC4748297; doi:10.1038/srep20583)
Supplement: Supplementary Information [file srep20583-s1.pdf]

Supplementary information

Temporal dynamics of choice behavior in rats and humans: an examination of pre- and post-choice latencies

Justine Fam[1]*, Fred Westbrook[1], Ehsan Arabzadeh[2, 3]
[1]School of Psychology, University of New South Wales, Australia
[2]Eccles Institute of Neuroscience, John Curtin School of Medical Research, Australian National University, Australia
[3]ARC Centre for Excellence for Integrative Brain Function, Australia
- j.fam@unsw.edu.au

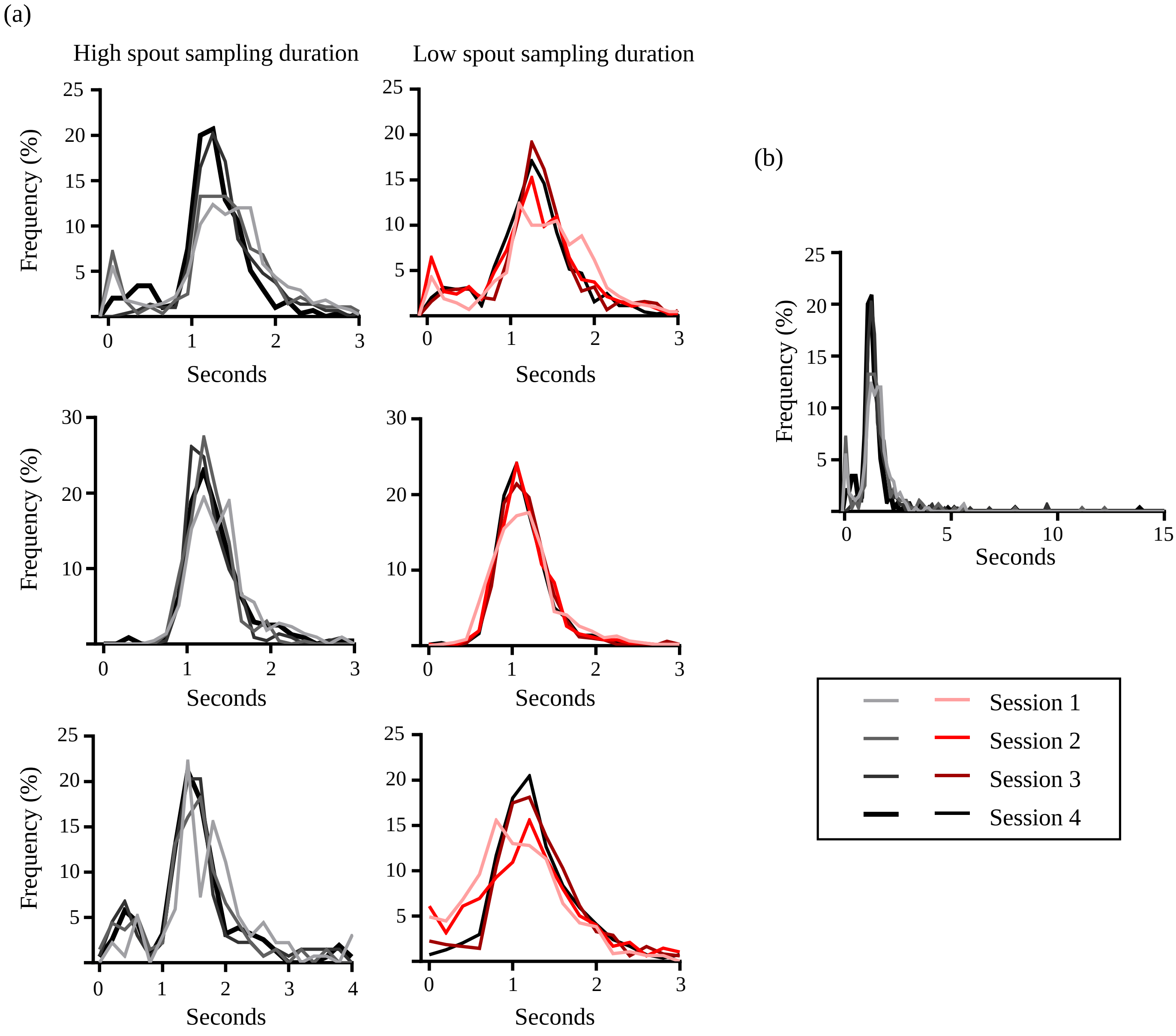

Supplementary Figure S1. (a) Distributions of spout sampling durations for Experiment 1_R, Group 60-40 (top), 70-30 (middle) and 80-20 (bottom) across the four experimental sessions. With the exception of Low spout sampling durations for Group 70-30, latencies from Session 1 have wide variable distributions compared to later sessions. In some cases, this variance is clearly evident as a bi-modal distribution and may reflect exploration of task requirements. In comparison, distributions from later experimental sessions are narrower and have more distinct uni-modal peaks. In this respect, Session 4 distributions overall show the least variance which likely reflects familiarity with the temporal structure of the task that could obscure effects of reward contingencies on behavior. For example, by the fourth session, rats would be familiar that the reward delays are not different between High and Low. For these latency figures, the x-axes have been truncated to focus on the bulk of the distribution, but **(b)** shows an example of a full distribution, with pronounced positive skew. This example shows the data for Group 60-40 High spout sampling durations.

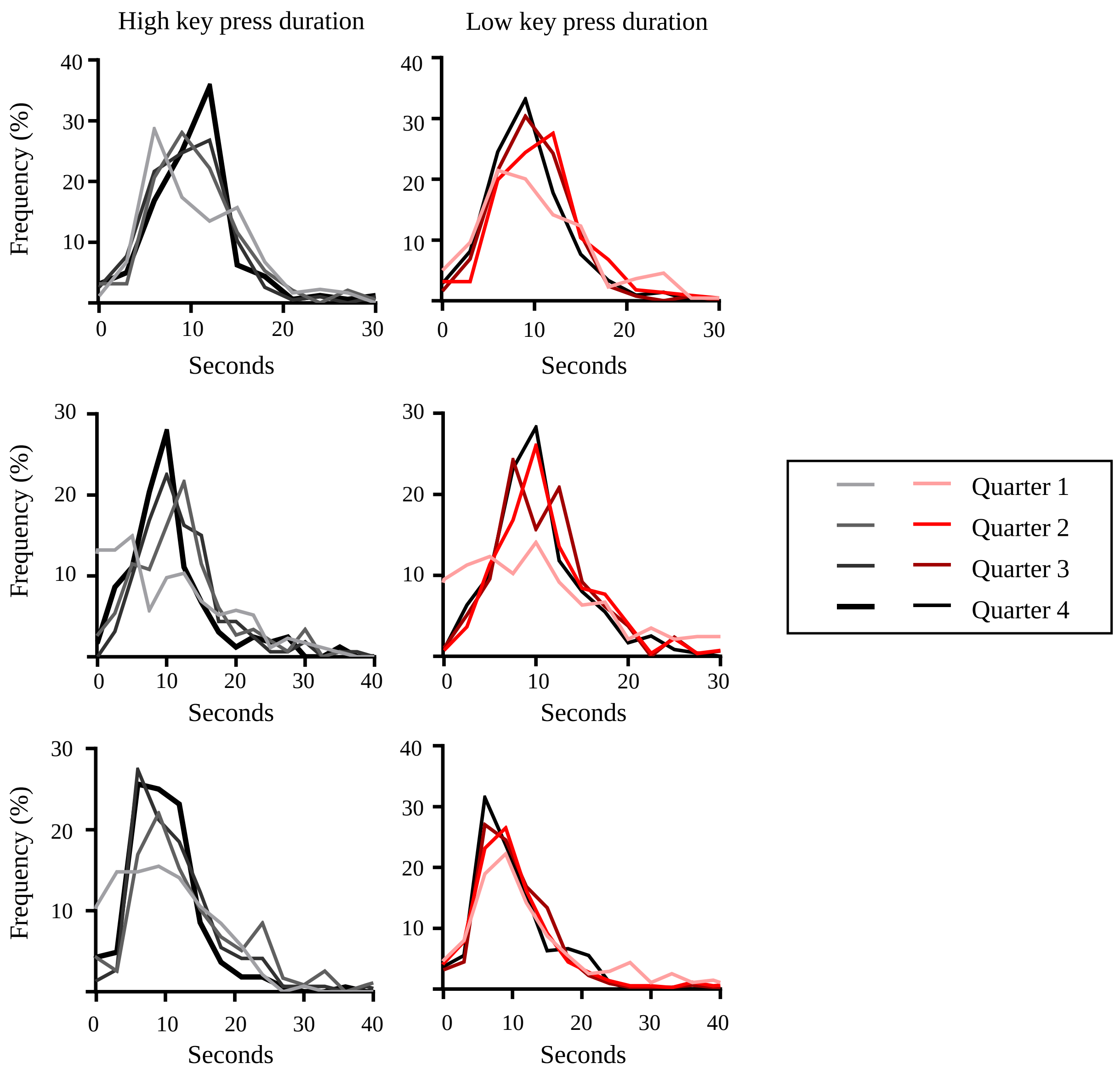

Supplementary Figure S2. Distributions of key press durations for Experiment 1_H,Group 60-40 (top), 70-30 (middle) and 80-20 (bottom) across the four quarters of the experimental session. Latencies from Quarter 1 have wide and variable distributions compared to later quarters. In some cases, this variance is clearly evident as a bi-modal distribution and may reflect exploration of task requirements. Quarter 4 distributions show the least variance which likely reflects familiarity with the temporal structure of the task that could obscure effects of reward contingencies on behavior. For example, by the last quarter, participants would be familiar that reward delays are not different between High and Low.

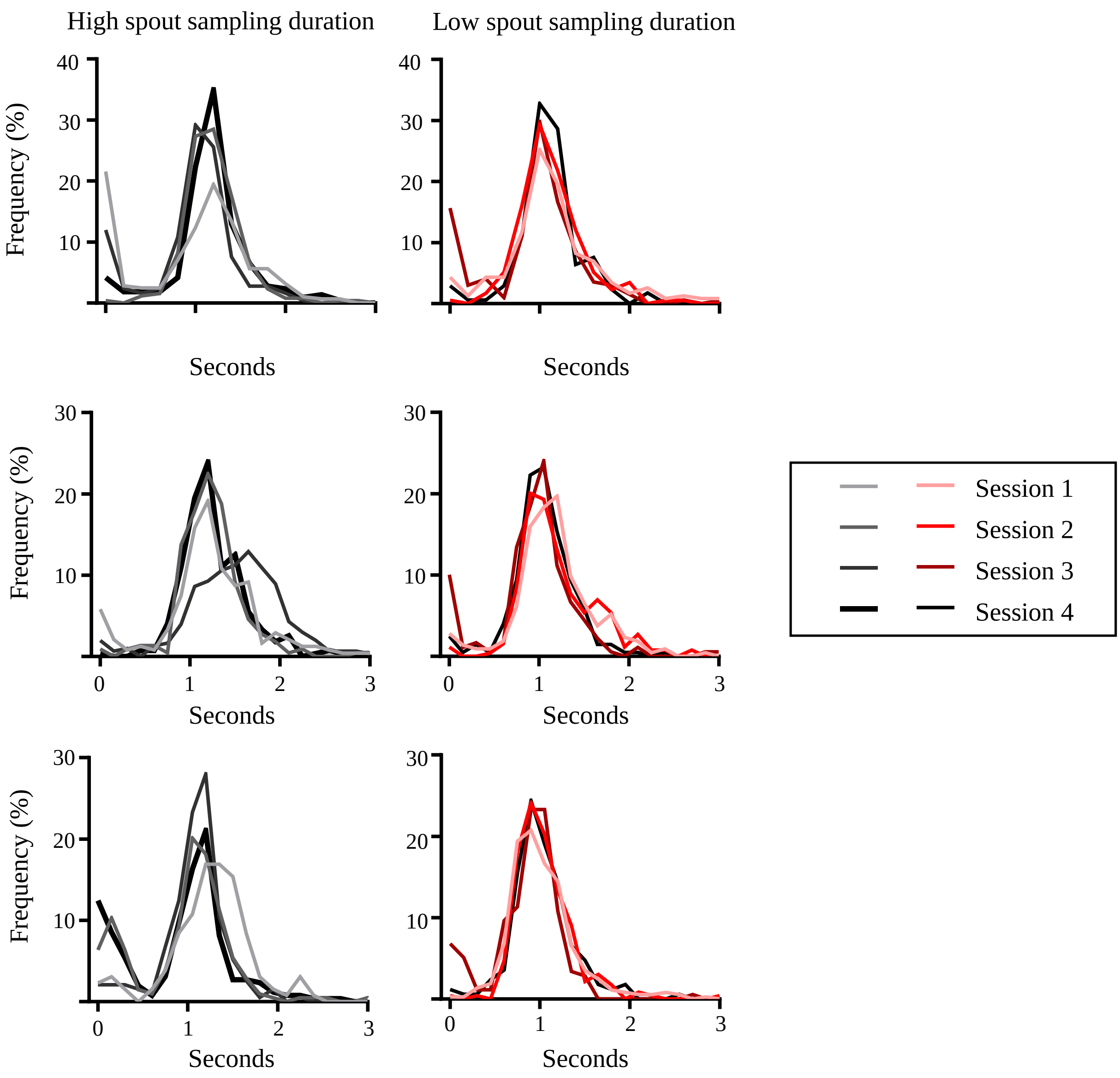

Supplementary Figure S3. Distributions of spout sampling durations for Experiment 2_R, Group 60-40 (top), 70-30 (middle) and 80-20 (bottom) across the four experimental sessions. For 60-40 Low, 70-30 High and 80-20 Low, latencies from Session 1 have wide and variable distributions compared to later sessions.

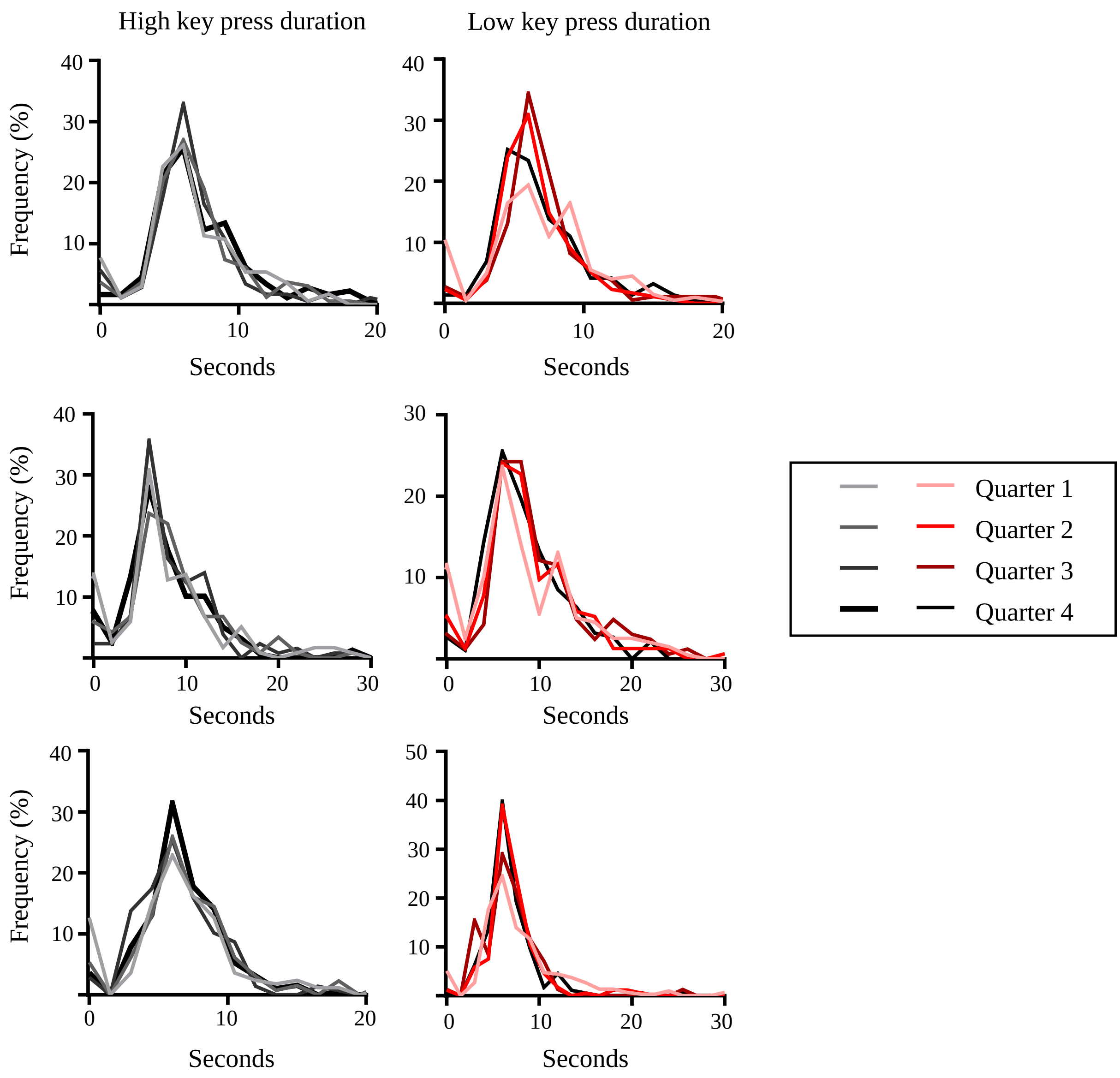

Supplementary Figure S4. Distributions of key press durations for Experiment 2_H,Group 60-40 (top), 70-30 (middle) and 80-20 (bottom) across the four quarters of the experimental session. Overall, latencies from Quarter 1 have wide and variable distributions compared to later quarters. In some cases, this variance is evident as a bi-modal distribution and may reflect exploration of task requirements. Quarter 4 distributions show the least variance which likely reflects familiarity with the temporal structure of the task that could obscure effects of reward contingencies on behavior. For example, by the last quarter, participants would be familiar that reward delays are not different between High and Low.

Supplementary Table S1. Descriptive statistics for latency variables for Experiment 1_R

Variable	Mean	Median	SD	SEM	Skewness*	Excess kurtosis**
60-40 High cue response time	1.01	.75	1.43	.04	11.51	203.9
60-40 Low cue response time	1.07	.81	1.12	.03	6.98	62.83
60-40 High spout sampling duration	1.65	1.38	1.49	.06	7.07	75.34
60-40 Low spout sampling duration	1.40	1.29	.97	.03	4.63	40.64
70-30 High cue response time	.79	.76	.25	.01	4.289	40.68
70-30 Low cue response time	1.09	.92	.93	.02	15.57	347.2
70-30 High spout sampling duration	1.51	1.25	1.31	.06	8.19	93.76
70-30 Low spout sampling duration	1.28	1.11	1.35	.04	14.66	262.5
80-20 High cue response time	.84	.75	.79	.02	12.18	187.7
80-20 Low cue response time	2.15	1.28	2.40	.07	7.87	93.8
80-20 High spout sampling duration	1.76	1.51	1.49	.09	4.092	25.86
80-20 Low spout sampling duration	1.59	1.24	1.96	.06	6.31	51.95

*skewness values between -.5 and .5 indicate that the distributions are approximately symmetric [1]
** a normal distribution is associated with excess kurtosis values of 0 [2]

Supplementary Table S2. Descriptive statistics for latency variables for Experiment 1_H

Variable	Mean	Median	SD	SEM	Skewness*	Excess kurtosis**
60-40 High cue response time	.87	.79	.96	.04	17.29	388.8
60-40 Low cue response time	.95	.85	.88	.03	7.78	84.15
60-40 High key press duration	10.47	9.77	5.42	.28	1.49	4.54
60-40 Low key press duration	10.27	10.07	4.65	.21	.93	2.70
70-30 High cue response time	.83	.68	.71	.03	6.70	67.10
70-30 Low cue response time	.86	.80	.55	.02	3.54	22.83
70-30 High key press duration	12.52	11.27	8.93	.51	4.90	44.71
70-30 Low key press duration	11.67	10.41	7.20	.30	2.92	17.16
80-20 High cue response time	.92	.88	.64	.02	12.52	238.7
80-20 Low cue response time	.96	.89	.49	.02	2.93	13.3
80-20 High key press duration	12..51	10.59	7.90	.49	1.79	5.17
80-20 Low key press duration	10.54	9.05	6.88	.27	2.34	9.28

*skewness values between -.5 and .5 indicate that the distributions are approximately symmetric [1]
** a normal distribution is associated with excess kurtosis values of 0 [2]

Supplementary Table S3. Descriptive statistics for latency variables for Experiment 2_R

Variable	Mean	Median	SD	SEM	Skewness*	Excess kurtosis**
60-40 High choice execution latency	.59	.48	2.37	.06	36.32	1406
60-40 Low choice execution latency	.50	.42	.67	.02	25.30	729.5
60-40 High spout sampling duration	1.19	1.10	1.15	.05	13.14	229.7
60-40 Low spout sampling duration	1.08	1.04	.67	.04	4.54	42.65
70-30 High choice execution latency	.51	.42	.90	.02	31.53	1207
70-30 Low choice execution latency	.56	.49	0.70	.02	21.76	566.6
70-30 High spout sampling duration	1.54	1.37	1.46	.08	15.01	285.2
70-30 Low spout sampling duration	1.31	1.06	1.70	.06	9.10	101.1
80-20 High choice execution latency	.50	.39	1.33	.03	33.1	1293
80-20 Low choice execution latency	.49	.39	.79	.03	13.69	211.9
80-20 High spout sampling duration	1.01	1.09	.46	.02	-.32	1.21
80-20 Low spout sampling duration	1.01	.96	.70	.03	8.94	113.6

*skewness values between -.5 and .5 indicate that the distributions are approximately symmetric [1]
** a normal distribution is associated with excess kurtosis values of 0 [2]

Supplementary Table S4. Descriptive statistics for latency variables for Experiment 2_H

Variable	Mean	Median	SD	SEM	Skewness*	Excess kurtosis **
60-40 High choice execution latency	.45	.32	.46	.02	5.56	49.02
60-40 Low choice execution latency	.45	.32	.52	.02	9.47	146.7
60-40 High key press duration	7.43	6.23	5.97	.32	6.24	62.57
60-40 Low key press duration	7.17	6.16	3.94	.21	2.32	8.35
70-30 High choice execution latency	.47	.35	.36	.01	3.33	23.10
70-30 Low choice execution latency	.51	.43	.37	.02	2.05	7.17
70-30 High key press duration	8.97	7.48	6.50	.41	3.98	28.40
70-30 Low key press duration	9.42	7.93	5.33	.30	1.48	3.98
80-20 High choice execution latency	.46	.37	.56	.02	10.91	166.2
80-20 Low choice execution latency	.55	.44	.49	.02	6.10	56.86
80-20 High key press duration	7.94	6.53	7.58	.46	4.47	23.99
80-20 Low key press duration	7.61	6.48	5.82	.32	4.44	23.42

*skewness values between -.5 and .5 indicate that the distributions are approximately symmetric [1]
** a normal distribution is associated with excess kurtosis values of 0 [2]

Supplementary Table S5. Spearman's rho for the relationship between pre- and post-choice latencies across time

Time point	*r*	*p*	Time point	*r*	*p*
Experiment 1R			Experiment 1H		
60-40 Session 1	-.08	.04	60-40 Quarter 1	-.04	.45
60-40 Session 2	-.12	.003	60-40 Quarter 2	.10	.04
60-40 Session 3	-.14	<.0001	60-40 Quarter 3	.02	.75
60-40 Session 4	-.11	<.0001	60-40 Quarter 4	.12	.02
70-30 Session 1	-.05	.24	70-30 Quarter 1	-.31	<.0001
70-30 Session 2	-.11	.002	70-30 Quarter 2	-.05	.01
70-30 Session 3	-.16	<.0001	70-30 Quarter 3	-.09	.01
70-30 Session 4	-.18	<.0001	70-30 Quarter 4	.01	.91
80-20 Session 1	-.20	<.0001	80-20 Quarter 1	.02	.73
80-20 Session 2	-.21	<.0001	80-20 Quarter 2	.30	<.0001
80-20 Session 3	-.13	.01	80-20 Quarter 3	.31	<.0001
80-20 Session 4	-.02	.002	80-20 Quarter 4	.24	<.0001
Experiment 2R			Experiment 2H		
60-40 Session 1	.01	.85	60-40 Quarter 1	-.11	.04
60-40 Session 2	-.05	.26	60-40 Quarter 2	.20	<.0001
60-40 Session 3	.13	.005	60-40 Quarter 3	-.11	.04
60-40 Session 4	-.12	.02	60-40 Quarter 4	-.04	.41
70-30 Session 1	-.22	<.0001	70-30 Quarter 1	-.16	.005
70-30 Session 2	-.10	.06	70-30 Quarter 2	.06	.34
70-30 Session 3	-.25	<.0001	70-30 Quarter 3	.38	<.0001
70-30 Session 4	-.17	<.0001	70-30 Quarter 4	.36	<.0001
80-20 Session 1	.04	.39	80-20 Quarter 1	-.02	.63
80-20 Session 2	.06	.20	80-20 Quarter 2	.09	.0005
80-20 Session 3	.12	.02	80-20 Quarter 3	.10	.001
80-20 Session 4	.12	.01	80-20 Quarter 4	.05	.33

References

1. Bulmer, M. G. *Principles of statistics*. 61-65 (Dover Publications, 1979)
2. Balanda, K. P., & MacGillivray, H. L. *Kurtosis: a critical review*. *Am Stat*, **42(2)**, 111-19 (1988) .
